# Cocaine

**Published:** 1886-01

**Authors:** C. H. Bushong

**Affiliations:** 337 W. 50th St.


### COCAINE.

BY C. H. BUSHONG, M .D.

Most races have some domestic drink. The Chinese have tea, the Arabs have coffee, the Brazillians also have coffee, and guarana (the latter was the drink of the native Indians) and the Peruvians have coca or cuca.

Of these beverages, all except the last, have the same active principal, called caffeine, in varying proportions. The active principle of coca is called *cocaine*, and it differs in most of its properties from caffeine.

The tea of coca tastes much like the Chinese tea, with which we are all familiar, and when taken in this form, its effects are that of a mild stimulant It is made by steeping the leaves of the plant, just as our tea is made.

These leaves are also chewed by the natives, much as Americans chew tobacco, and in this form the peculiar properties begin to show themselves. The mountaineers of the Andes use it when they are enduring hardships of any kind. They know that it conceals the sensations of fatigue, hunger and drowsiness. By chewing coca leaves they can go longer without rest, food or sleep than they otherwise could, and they seem to suffer little from the effects that are usually found to follow such privations.

These properties of coca have been known to medical men for years, yet it seemed to be a remedy of minor importance, as other and better known drugs seemed to possess all the medicinal properties of this one, and were considered more efficacious.

It was late in the summer of 1884 that Koller announced his discovery. He was a medical student in Vienna. While experimenting in the laboratory there he accidentally learned that a solution of the hydrochlorate (muriate) of cocaine, if dropped into the eye, in a few minutes caused a complete loss of feeling in that organ. This condition of localized anaesthesia he found to be of short duration, lasting about twenty minutes. After these effects had passed away, the eye at once regained its normal condition, and seemed to suffer no ill effects from the application, even if it was repeated several times.

In a very short time an account of this newly found property of cocaine had reached most of the civilized world, and experiments were made in many places. Its importance for cases of minor surgery was at once recognized by the leading men of the profession. The surgeon can operate in the brief period of loss of sensation, and thus avoid the dangers attending the use of a general anaesthetic, as ether or chloroform. He can, also, dispense with at least one assistant. The patient is also saved the very disagreeable after effects so general from ether. Many nervous and timid persons will find small operations robbed of their chief terrors when they can have them done without pain and yet be in full possession of all their mental faculties. The necessity of a period of forced unconsciousness is much dreaded by many.

Much time is saved to both the patient and surgeon by the use of cocaine. There is no long period of waiting for him to get "under the influence" before operating, and also no waiting for him to "come to" after it.

I have seen children, with the aid of cocaine, sit through that usually painful operation for squint without wincing, and have heard them remark that "it did not hurt at all."

Since Koller's announcement of his discovery, cocaine has been tried for many other operations, and much has been added to our knowledge of its action. It has been learned that by controlling the circulation properly, the period of loss of sensation may be prolonged to any desired length of time This has greatly enlarged the field of its usefulness. The former brief period did not give time for working with deliberation and thoroughness, in operations of greater magnitude, and these are important to the success of an operation.

An article on cocaine would be incomplete without mention of the dangers from its use, or rather its abuse. One case has been reported in which death ensued from the hypodermatic administration of what is generally considered a perfectly safe dose. This shows that persons vary in their susceptibility to its influence.

Several cases of the formation of a cocaine habit have been noted, and they seem to indicate that this drug exceeds morphine in its enslaving power over its victim, and that its influences are more debasing. It is said to destroy all moral and social instincts, making its user a mere animal, with no desire beyond those of self and selfish gratification.

More observation is necessary to establish these points, but enough has been seen and is known of the evil effects from the abuse of cocaine, to justify its being sold under the same restrictions as morphine, and when sold, it should have a "poison" label attached. It is most dangerous when injected under the skin.

Many people believe that cocaine is obtained from cocoa. This is a very natural mistake resulting from the similarity in the names of these two articles. They have little resemblance except in name. This will appear from a brief comparison of the two. Cocaine is a derivative of *Erythroxylon coca*, while cocoa is obtained from *Theobroma*

*cocoa.* The Erythroxylon is a shrub, the Theobroma is a tree ; the cocoa-nut tree. Further, cocaine is obtained from the *leaves* while cocoa, or chocolate, is the prepared *kernel* of the *nuts*. Finally, cocaine is a dangerous though very useful drug, but cocoa is a harmless and nourishing drink, and when properly prepared is more wholesome than tea or coffee.

337 W. 50th St.,

